# Correction: An armoured marine reptile from the Early Triassic of South China and its phylogenetic and evolutionary implications

**DOI:** 10.7554/eLife.107998

**Published:** 2025-06-11

**Authors:** Andrzej S Wolniewicz, Yuefeng Shen, Qiang Li, Yuanyuan Sun, Yu Qiao, Yajie Chen, Yi-Wei Hu, Jun Liu

**Keywords:** Other

Wolniewicz AS, Shen Y, Li Q, Sun Y, Qiao Y, Chen Y, Hu Y-W, Liu J. 2023. An armoured marine reptile from the Early Triassic of South China and its phylogenetic and evolutionary implications. *eLife* **12**:e83163. doi: 10.7554/eLife.83163.

Published 8 August 2023

It was recently noticed that the originally published version of Figure 2 included an error in the labelling of the skeletal reconstruction (Fig. 2B). The right hindlimb was mistakenly indicated as preserved, whereas it is in fact the left hindlimb that is preserved in the fossil specimen (HFUT YZSB-19-109; Fig. 2A). A corrected version of the skeletal reconstruction is now provided, with the left hindlimb correctly labelled as preserved.

The corrected Figure 2 (with panel B updated) is shown here:

**Figure 1:**
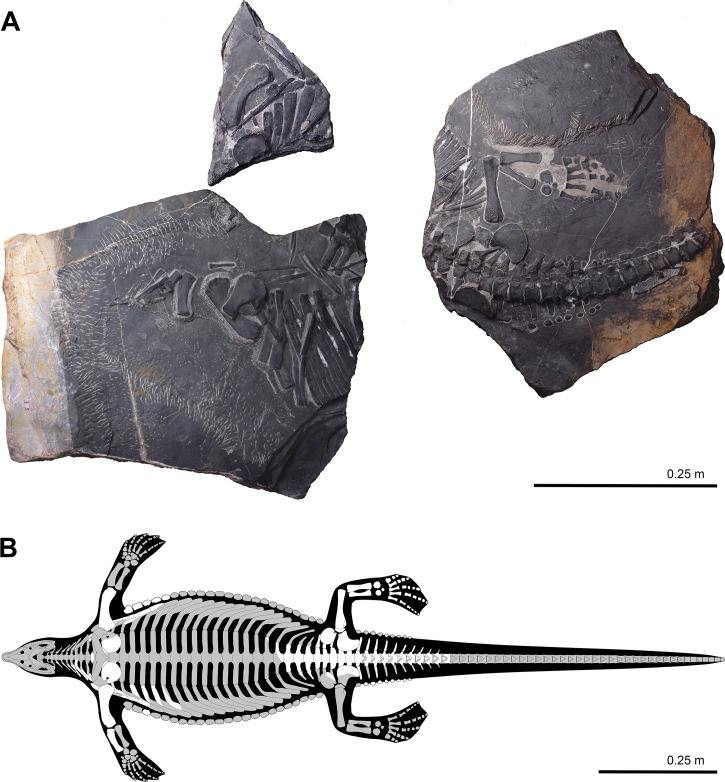


The originally published Figure 2 is shown for reference:

**Figure 2:**
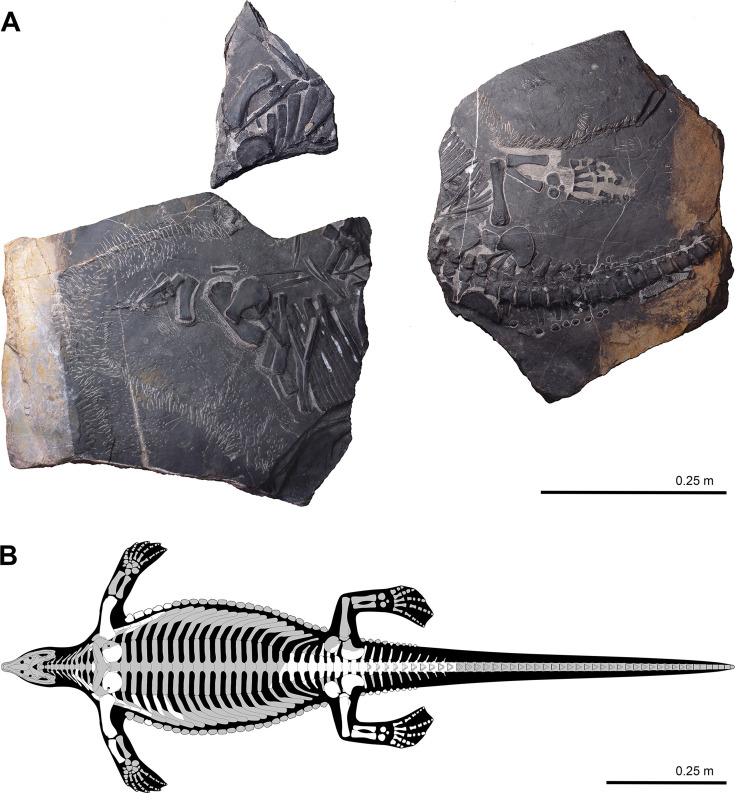


The article has been corrected accordingly.

